# Acute Sin Nombre hantavirus infection without pulmonary syndrome, United States.

**DOI:** 10.3201/eid0505.990512

**Published:** 1999

**Authors:** P T Kitsutani, R W Denton, C L Fritz, R A Murray, R L Todd, W J Pape, J Wyatt Frampton, J C Young, A S Khan, C J Peters, T G Ksiazek

**Affiliations:** Centers for Disease Control and Prevention, Atlanta, Georgia 30333, USA. pdk8@cdc.gov

## Abstract

Hantavirus pulmonary syndrome (HPS) occurs in most infections with Sin Nombre virus and other North American hantaviruses. We report five cases of acute hantavirus infection that did not fit the HPS case definition. The patients had characteristic prodromal symptoms without severe pulmonary involvement. These cases suggest that surveillance for HPS may need to be expanded.


### Acute Sin Nombre Hantavirus Infection without Pulmonary Syndrome, United States

**Paul T. Kitsutani,* Robert W. Denton,† Curtis L. Fritz,‡ Robert A. Murray,§ Randall L. Todd,¶# W. John Pape,** J. Wyatt Frampton,†† Joni C. Young,* Ali S. Khan,* Clarence J. Peters,* and Thomas G. Ksiazek***

*Centers for Disease Control and Prevention, Atlanta, Georgia, USA; †Bishop, California, USA; ‡California Department of Health Services, Sacramento, California, USA; §California Department of Health Services, Berkeley, California, USA; ¶Bureau of Disease Control and Intervention Services, Carson City, Nevada, USA; #Nevada State Health Division, Carson City, Nevada, USA; **Colorado Department of Public Health & Environment, Denver, Colorado, USA; and ††Utah Department of Health, Salt Lake City, Utah, USA

Hantavirus pulmonary syndrome (HPS) occurs in most infections with Sin Nombre virus and other North American hantaviruses. We report five cases of acute hantavirus infection that did not fit the HPS case definition. The patients had characteristic prodromal symptoms without severe pulmonary involvement. These cases suggest that surveillance for HPS may need to be expanded.

Hantavirus pulmonary syndrome (HPS) is an emerging infectious disease, often characterized by rapid, dramatic clinical progression and high case-fatality rates. Most cases in the United States are caused by Sin Nombre virus (SNV); like other viruses causing HPS, SNV has a single rodent host belonging to the subfamily *Sigmodontinae* (1). The virus is transmitted to humans through inhalation of aerosolized feces, urine, or saliva from infected rodents.

Since the initial outbreak in the Four Corners region in 1993 (2), 217 cases of HPS were reported in the United States as of May 28, 1999; 32 of these occurred before May 1993. These cases have provided information on the clinical symptoms, disease progression, and laboratory characteristics of HPS. An incubation period of 2 to 3 weeks is typically followed by high fever, myalgia, headache, fatigue, and gastrointestinal symptoms (3,4). This phase is followed 4 to 6 days later by abrupt onset of dyspnea and hypoxia, typically associated with noncardiac pulmonary edema and respiratory failure, requiring hospitalization and intensive-care management (4,5). Hypotension or shock with myocardial depression is present in most patients; renal dysfunction of varying severity is sometimes observed. Common laboratory findings include elevated hematocrit, leukocytosis with left shift and immature myelocytes and immunoblasts, and thrombocytopenia (6,7). The diagnosis is confirmed by serologic testing for hantavirus SNV immunoglobulin (Ig)M and IgG, although reverse transcriptase polymerase chain reaction (RT-PCR) or immunohistochemical analysis (IHC) can also be done.

In a study of the prevalence of SNV antibody in patients who had mild febrile illness during the 1993 HPS outbreak, asymptomatic and mild infections were uncommon (8). This observation contrasts with reports of hantaviruses that cause hemorrhagic fever with renal syndrome; mild disease can occur after infection with Hantaan virus and is predominately associated with Puumala virus infections (9,10). Since May 1993, five persons with mild acute HPS illness have

Address for correspondence: Paul T. Kitsutani, Centers for Disease Control and Prevention, 1600 Clifton Road, Mail Stop A26, Atlanta, GA 30333, USA; fax: 404-639-1509; e-mail: pdk8@cdc.gov.

been identified; one was a 4-year-old boy whose case has been described (11). We describe the other four cases, two of which were detected in 1998 and one in 1999.

HPS is clinically defined as a febrile illness (temperature >38.3°C) with bilateral diffuse infiltrates that cause respiratory compromise requiring supplemental oxygen within 72 hours of hospitalization (12). A case may also be defined postmortem as an unexplained, fatal respiratory illness, with noncardiogenic pulmonary edema of unknown cause. Clinically suspected cases are confirmed by fulfilling one of three criteria at a reference laboratory: detection of hantavirus-specific IgM or rising titers of IgG antibodies, or hantavirus-specific RNA sequence by RT-PCR, or hantavirus antigens in tissues by IHC.

These four atypical cases were identified through the National HPS Surveillance System, although they did not meet the clinical criteria for HPS. In Patients 2 and 3, infection with SNV was suspected early in the illness, and sera were tested promptly for hantavirus antibodies. Serum from Patient 1 was tested for SNV antibody retrospectively, after a friend with a common exposure history was diagnosed with HPS. Acute- and convalescent-phase sera from each patient were also tested at CDC for hantavirus IgM and IgG antibodies by using a panel of prototypic hantavirus strains (13). Cases were confirmed as acute SNV infections if there were substantial titers of anti-SNV IgM and either substantial acute-phase titers of IgG or a fourfold rise in convalescent-phase IgG titers.

### Case Report 1

A 38-year-old previously healthy man from Nevada visited a local emergency room in October 1993 with a 3-day history of fever, headache, fatigue, malaise, dizziness, progressive myalgia, dry cough, and shortness of breath (Table). His temperature was 38.3°C, pulse 103 per minute, and blood pressure 130/77 mm Hg. His oxygen saturation was 85% on room air, improving to 94% on 2 liters of oxygen by nasal cannula. Except for frontal sinus tenderness, his physical examination was otherwise unremarkable.

Approximately 2 weeks later, a friend of the patient's died of a respiratory illness diagnosed as HPS. A case investigation of all household and social contacts led retrospectively to the diagnosis of acute hantavirus infection in the patient, as demonstrated by positive SNV IgM and IgG titers. The patient and his friend had worked together at a ranch in rural Nevada, where they slept for 2 days in a rodent-infested guest house (14).

### Case Report 2

A 36-year-old woman from California visited her physician in July 1998 with a 2-day history of fever, headache, and malaise. Her temperature was 37.5°C, pulse 130 per min, and blood pressure 90/60/mm Hg. Physical examination was unremarkable.

Two days later, she visited the emergency room for the same complaints, as well as myalgia, dry cough accompanied by substernal burning and pain, sore throat, vomiting, and photophobia (Table). She was not in acute respiratory distress. An infectious mononucleosis test was positive. She was diagnosed with a viral syndrome probably secondary to mononucleosis and dehydration, treated with intravenous fluids, and discharged. Her clinical course improved without hospitalization.

The patient worked as a registered nurse and lived on a ranch. She had no history of recent travel. She reported three exposures to rodent excreta in the month before becoming ill, twice while cleaning a barn and once while cleaning her mobile home. Because of this history, she was tested for SNV antibodies on day 2 of illness. This acute-phase serum, as well as a convalescent-phase serum, tested positive for both SNV IgM and IgG antibodies.

### Case Report 3

A previously healthy 19-year-old man visited a local Colorado emergency room in June 1999 with a 2-day history of fever, chills, myalgia, nausea, and vomiting, but no shortness of breath. His vital signs included a temperature of 39.5°C, pulse of 93 per minute, blood pressure 114/71 mm Hg, and oxygen saturation of 89.9% on room air. A platelet count was 96,000/mm$^3$. A chest X-ray was unremarkable. A diagnosis of HPS was suspected because of a history of rodent exposure in the community, with two recent fatal cases, but the patient refused hospitalization.

The patient was admitted 2 days later after his initial serum specimen was noted to have SNV IgM and IgG antibodies. He felt better, although he remained febrile and had developed a slight cough. A repeat chest X-ray was initially reported as normal but was retrospectively read

Table. Characteristics of five acute cases of Sin Nombre Virus infection without pulmonary syndrome, 1993–1999

| | Case 1, 1993 | Case 2, 1998 | Case 3, 1999 | Case 4, 1998[a] | Case 5, 1993[b] |
|---|---|---|---|---|---|
| Age (yr) | 38 | 36 | 19 | 32 | 4 |
| Sex | M | F | M | M | M |
| Race | White | White | White | White | American Indian |
| State | Nevada | California | Colorado | Utah | New Mexico |
| Hospitalized | No | No | Yes | Yes | No |
| Symptoms | | | | | |
| Fever | +[c] | + | + | + | + |
| Headache | + | + | - | + | - |
| Malaise | + | + | - | - | - |
| Myalgia | + | + | + | + | - |
| Cough | + | + | + | - | + |
| Shortness of breath | + | - | - | - | - |
| Chest/substernal pain | - | + | - | - | - |
| Sore throat | - | + | - | - | NR[d] |
| Nausea/vomiting | + | + | + | - | NR |
| Dizziness | + | - | + | - | NR |
| Photophobia | - | + | - | | |
| Abdominal pain | - | - | - | - | NR |
| Diarrhea | - | - | - | - | NR |
| Arthralgia | - | - | - | + | NR |
| Vital signs | | | | | |
| Max temp (°F) | 102.5 | 104.0 | 103.1 | 102.8 | 100.6 |
| Blood pressure | normal | normal | normal | normal | NR |
| Lowest $O_2$ sat. (RA[e]) | 85% | 94% | 89.9% | 94% | NR |
| Laboratory results | | | | | |
| Highest Hct (%) | 47.0 | 44.7 | 50.2 | 44.4 | 40.2 |
| Highest WBC | 9,100 | 8,000 | 10,200 | 7,300 | NR[f] |
| % seg. neutrophils | 68 | 40 | 39 | 88 | |
| % bands | 9 | 30 | 24 | | |
| % lymphocytes | 19 | 14 | 20 | 7 | |
| % atypical lymphocytes | NR | 4 | NR | NR | |
| Lowest platelet (/mm$^3$) | 127,000 | 115,000 | 28,000 | 163,000 | NR[g] |
| Highest SGOT[h] (U/L) | NR | 81 | NR | 26 | NR |
| Highest LDH[i] (U/L) | NR | 337 | 488 | 240 | NR |
| Lowest albumin (g/dL) | NR | 3.1 | NR | 4.3 | NR |
| Chest X-ray | 2-cm granuloma | normal | Mild left lower lobe infiltrate | normal | NR |
| Anti-SNV[j] antibody | | | | | |
| IgM | positive | positive | positive | positive | positive |
| IgG | positive | positive | positive | positive | positive |

[a]Obtained from Zavasky D-M, Hjelle B, Peterson M, et al. Acute infection with Sin Nombre hantavirus without pulmonary edema. Clin Infect Dis, in press.
[b]Obtained from Armstrong et al., 1995 (12).
[c]+, present; -, absent.
[d]NR = not recorded or obtained.
[e]RA = room air.
[f]Patient did not have leukocytosis.
[g]Patient did not have thrombocytopenia.
[h]SGOT = serum glutamic oxalacetic acid.
[i]LDH = lactic dehydrogenase.
[j]SNV = Sin Nombre virus.

as having a slight left lower lobe interstitial infiltrate. His symptoms gradually resolved and he was discharged 2 days after admission.

### Conclusions

These patients are among the first adults in the United States to have had acute SNV infections resulting in illnesses less severe than HPS; a fourth is being described elsewhere (Table). These patients had the characteristic HPS-like prodromal symptoms of high fever, headache, and mylagia. Some of the other typical features of HPS (malaise, nausea, vomiting, dizziness, cough, chest pain) were also observed. Patient 1 initially had signs and symptoms of pulmonary involvement, documented by low oxygen saturation. In contrast, Patients 2 and 3 did not have respiratory distress, although Patient 3 had one oxygen saturation measurement of 89.5% on room air. All four patients had normal lung findings on physical examination and characteristic diffuse bilateral interstitial edema was not seen on chest X-rays.

Several of the typical laboratory findings of HPS were noted, including a left shift on the white blood count differential, atypical lymphocytes, mildly elevated serum glutamic oxalacetic acid or lactic dehydrogenase, and low albumin. All the reported patients had unequivocal thrombocytopenia, and Patient 4 had a decreasing platelet count. The hematocrit of Patient 2 rose from 42.3% to 44.7% during her acute illness, then decreased to 36.3%, suggesting a period of substantial hemoconcentration, as seen in HPS.

All three patients became ill in areas of the United States where reservoirs of other known pathogenic U.S. hantaviruses are not found and where all RT-PCR-typed HPS cases have been caused by SNV.

During the initial 1993 outbreak, an intensive search for SNV IgM and IgG antibodies was conducted among household contacts of patients (15), as well as among patients with acute fever and myalgia resembling HPS prodromal symptoms (8). IgM antibodies reacting with SNV were not found in the study population, which suggests that mild acute hantavirus infections were uncommon.

The first case of mild SNV illness with positive SNV IgM and IgG antibodies was described in a 4-year-old boy who had upper respiratory infection symptoms and otitis media but no other abnormal laboratory findings (11).

Mild cases of HPS have been observed in patients who did not have severe pulmonary disease or respiratory failure. In addition, a few patients with HPS with an initial normal chest X-ray have been described (16). However, chest X-rays 24 to 48 hours later demonstrated interstitial or alveolar edema in all these patients. These cases of mild HPS must be distinguished from the three cases reported in this article, which had no or minimal radiologic pulmonary involvement.

It is unclear why severe respiratory distress, pulmonary edema, and hypotension or shock, the hallmarks of HPS, did not develop in these patients. Histopathologic and immunologic studies of acute HPS patients have shown antibodies, significant CD8 and CD4 T-lymphocyte activation, and lymphokine involvement, suggesting the hypothesis that HPS is an immunopathologic response to hantavirus infection (6,7,13,17,18). Patients with mild SNV illness may have a weaker immune response to the virus than patients whose illness progresses to HPS. In addition, integrins expressed on platelets and endothelial cells have recently been implicated as a vehicle for HPS-associated SNV and NY-1 cellular entry and pathogenicity (19). Physiologic or genetic variations in these receptor molecules may provide another potential explanation for differing hantavirus pathogenesis.

Virologic factors may also play a role in the development of mild illness. Like the hantaviruses that cause a clinical spectrum of hemorrhagic fever with renal syndrome, less pathogenic strains of SNV or other American hantaviruses may not yet have been characterized. Further virologic, molecular, and immunologic analyses of these and other cases may provide better insights into the pathophysiologic mechanisms of mild SNV disease.

Domestic exposure to rodent excreta continues to be a major risk factor for contracting HPS. Public health education of risk-reducing measures against hantavirus infection should remain a high priority. Moreover, SNV infection should be considered in the differential diagnoses of patients with nonspecific febrile illness and a history of possible exposure to rodents.

### Acknowledgments

The authors thank Daniel Safranek for the clinical details of Case 3 and Michael C. Peterson for a medical description of Case 4. We also thank Stuart T. Nichol and Pierre E. Rollin for their helpful review of this manuscript.

Dr. Kitsutani is an officer in CDC's Epidemic Intelligence Service.

### References

1. Monroe MC, Morzunov SP, Johnson AM, Bowen MD, Artsob H, Yates T, et al. Genetic diversity and distribution of *Peromyscus*-borne hantaviruses in North America. Emerg Infect Dis 1999;5:75-86.
2. Centers for Disease Control and Prevention. Outbreak of acute illness-southwestern United States, 1993. MMWR Morb Mortal Wkly Rep 1993;42:421-4.
3. Khan AS, Khabbaz RF, Armstrong LR, Holman RC, Bauer SP, Graber J, et al. Hantavirus pulmonary syndrome: the first 100 U.S. cases. J Infect Dis 1996;173:1297-303.
4. Duchin JS, Koster FT, Peters CJ, Simpson GL, Tempest B, Zaki SR, et al. Hantavirus pulmonary syndrome: a clinical description of 17 patients with a newly recognized disease. N Engl J Med 1994;330:949-55.
5. Hallin GW, Simpson SQ, Crowell RE, James DS, Koster FT, Mertz GJ, et al. Cardiopulmonary manifestations of hantavirus pulmonary syndrome. Crit Care Med 1996;24:252-8.
6. Zaki SR, Greer PW, Coffield LM, Goldsmith CS, Nolte KB, Foucar K, et al. Hantavirus pulmonary syndrome: pathogenesis of an emerging infectious disease. Am J Pathol 1995;146:552-79.
7. Nolte KB, Feddersen RM, Foucar K, Zaki SR, Koster FT, Madar D, et al. Hantavirus pulmonary syndrome in the United States: a pathological description of a disease caused by a new agent. Hum Pathol 1995;26:110-20.
8. Simonsen L, Dalton MJ, Breiman RF, Hennessy T, Umland ET, Sewell CM, et al. Evaluation of the magnitude of the 1993 hantavirus outbreak in the southwestern United States. J Infect Dis 1995;172:729-33.
9. Lee HW, van der Groen G. Hemorrhagic fever with renal syndrome. Prog Med Virol 1989;36:62-102.
10. Settergren B. Nephropathia epidemica (hemorrhagic fever with renal syndrome) in Scandinavia. Reviews of Infectious Diseases 1991;13:736-44.
11. Armstrong LR, Bryan RT, Sarisky J, Khan AS, Rowe T, Ettestad PJ, et al. Mild hantaviral disease caused by Sin Nombre virus in a four-year-old child. Pediatr Infect Dis J 1995;14:1108-10.
12. Centers for Disease Control and Prevention. Case definitions for infectious conditions under public health surveillance. MMWR Morb Mortal Wkly Rep 1997;46(RR-10):16.
13. Ksiazek TG, Peters CJ, Rollin PE, Zaki S, Nichol S, Spiropoulou C, et al. Identification of a new North American hantavirus that causes acute pulmonary insufficiency. Am J Trop Med Hyg 1995;52:117-23.
14. Wells RM, Young J, Williams RJ, Armstrong LR, Busico K, Khan AS, et al. Hantavirus transmission in the United States. Emerg Infect Dis 1997;3:361-5.
15. Zeitz PS, Butler JC, Cheek JE, Samuel MC, Childs JE, Shands LA, et al. A case-control study of hantavirus pulmonary syndrome during an outbreak in the southwestern United States. J Infect Dis 1995;171:864-70.
16. Ketai LH, Williamson MR, Telepak RJ, Levy H, Koster FT, Nolte KB, et al. Hantavirus pulmonary syndrome: radiographic findings in 16 patients. Radiology 1994;191:665-8.
17. Ennis FA, Cruz J, Spiropoulou CF, Waite D, Peters CJ, Nichol ST, et al. Hantavirus pulmonary syndrome: CD8+ and CD4+ cytotoxic T lymphocytes to epitopes on Sin Nombre virus nucleocapsid protein isolated during acute illness. Virology 1997;238:380-90.
18. Simpson SQ, Mapel V, Koster FT, Montoya J, Bice DE, Williams AJ. Evidence for lymphocyte activation in the hantavirus pulmonary syndrome. Chest 1995;106:97S.
19. Gavrilovskaya IN, Shepley M, Shaw R, Ginsberg MH, Mackow ER. ß3 integrins mediate the cellular entry of hantaviruses that cause respiratory failure. Proc Natl Acad Sci U S A 1998;95:7074-9.
